# Identifying Mothers with Postpartum Depression Early: Integrating Perinatal Mental Health Care into the Obstetric Setting

**DOI:** 10.5402/2011/309189

**Published:** 2011-09-15

**Authors:** Helen Chen, Jemie Wang, Ying Chia Ch'ng, Roshayati Mingoo, Theresa Lee, Julia Ong

**Affiliations:** Postnatal Depression Intervention Programme, Mental Wellness Service, Kandang Kerbau Women's and Children's Hospital (KKH), 100 Bukit Timah Road, Singapore 229899

## Abstract

With prevalence rates of postnatal depression (PND) as high as at least 7%, there was a need for early detection and intervention of postpartum mental illness amongst Singaporean mothers. This is a report on the first year results of our country's first PND Intervention Programme. The programme consists of two phases: (1) postpartum women were screened with the Edinburgh Postnatal Depression Scale and provided appropriate care plans; (2) individualized clinical intervention using a case management multidisciplinary team model. Screening for PND was generally acceptable, as 64% eligible women participated voluntarily. Nine percent (126) were identified as probable cases from 1369 women. Forty-one women accepted intervention and achieved 78% reduction in the EPDS symptom scores to below the cutoff of 13, 76% had improvement in GAF functioning scores, and 68% had improved health quality scores. Preliminary results are promising, and this intervention model can be replicated.

## 1. Introduction

The prevalence of perinatal depression in Singapore is about 12% for antepartum depression and about 7% for postpartum depression [1]. Peripartum depressive symptomatology is seen in up to 1 in 5 pregnant women, although not all amounting to major depression [2]. Adverse outcomes include unfavourable parenting practices [3], impaired mother-infant bonding, impaired intellectual and emotional development of the infant [4], and tragically, maternal suicide and infanticide [5]. 

In the USA, a number of comprehensive and integrated services have been established to cater maternal mental health [6, 7]. *Beyondblue*, the Australian National Depression Initiative also includes a programme targeting the postpartum depression [8]. Without any previous local programme, the study team set out to develop a pilot programme addressing postpartum depression.

The programme, funded by the health ministry, ran at the Kendang Kerbau Women's and Children's Hospital (KKH), which handles some 12,000 deliveries annually, representing about a third of the national births. The psychiatric service was established in 2006 to provide liaison consultative and outpatient psychiatric services, and referral had largely relied on the interests and the limited resources of the frontline obstetric professionals who were understandably more focused on obstetric issues. An integrated service was needed to provide accessible screening and early intervention for postpartum depression. Whilst intervening antenatally is ideal as antepartum depression is an important risk factor for postpartum depression [9, 10], with the limited resources logistically, the study team focused efforts on:

- screening and early detection of postpartum depression and severe postpartum mental illness,
- managing postpartum depression early using a case management multidisciplinary model to improve the outcomes.

We report here the results of the first year of the programme.

## 2. Materials and Methods

This is a prospective cohort study of postpartum women, conducted at KKH between 1 April 2008 to 31 March 2009, approved by the KKH Institutional Review Board, and funded by the Ministry of Health and the KKH Research Small Grant.

### 2.1. Target Population

Participants were recruited from two obstetric outpatient clinics handling about 2000 postpartum women annually, at 2 weeks to 6 months postdelivery. Excluded were (i) adolescent mothers (<18 yrs), as they were already cared for on an established medical social work programme, which included emotional support, (ii) women with stillbirth or early neonatal loss, (iii) those already receiving psychiatric treatment and close monitoring since the antepartum.

### 2.2. Screening Procedure

Participants were interviewed by the perinatal mental health case managers, using the Edinburgh Postnatal Depression Scale (EPDS) [11] as a guide. The widely used 10-item EPDS is an easy-to-use tool and reliable self-reported scale. It has been validated in many countries, including the Chinese version in Hong Kong [12] and our Singaporean peripartum population [1]. We made a minor modification to the EPDS, to include the qualifier of symptoms “in the past one week,” as in a pilot run we noted that local women tended to misinterpret the questions as referring to the general past. Recognising that the groups most at risk of tragic outcomes are those suffering from psychotic disorders or who have infanticidal impulses, we drew from earlier research by Kok et al. [13] to include three additional questions (see the Appendix). The best use of the EPDS is for routine postnatal screening, and we used a threshold of 13 to identify women likely to suffer from major depression, as determined by the Edinburgh and Cambridge researchers in a community sample [11, 14]. The interviews were conducted in a private setting, with individualised support and counseling provided immediately. This was much needed, as there is no system of universal midwifery support, unlike in the UK.

The high scorers (EPDS > 12), or those who answered yes to any of the three additional questions, were offered a psychiatric consultation, thus entering the intervention phase. 

**Figure 1:**
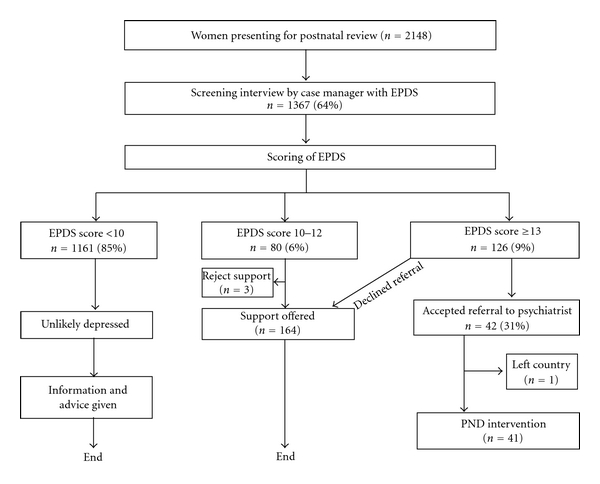
Process flow of the Postnatal Depression Intervention Programme.

Those scoring borderline (EPDS 10–12) were mostly found to have minor problems or difficulties related to adjustment to motherhood, not amounting to clinical depression, and they were provided counselling and offered follow-up phone review and/or counselling by the assigned case manager. The case manager would then advise if there was a need to see the psychiatrist, if the difficulties worsened or continued, or be directed to community resources as was appropriate. For high scorers that declined to the intervention, the assigned case managers would conduct phone followup to check on their progress. The process flow of the programme is detailed in Figure 1.

### 2.3. The Intervention Programme

Early intervention for the high scorers included full psychiatric assessments, with supportive counseling, psychoeducation, and problem-solving focused counseling incorporating the principles of interpersonal and cognitive behavioural therapy (Table 1). Antidepressant medication for cases was recommended for those with depression of moderate severity. For breastfeeding women, tricyclic antidepressants were the firstline drugs considered, as recommended in the UK guidelines [15] whilst those not nursing were given various drugs as determined clinically by the managing psychiatrist. A case management model was used, providing integrated, individualized, and continuous care from screening to intervention [16]. Patients were encouraged to join the support group facilitated by the case managers, as postpartum depression peer support has been shown to be beneficial [17]. For women with more entrenched psychological difficulties, formal psychotherapy was provided by the multidisciplinary team psychologist. Women with mother-infant bonding difficulties were referred to the occupational therapy for baby massage, whilst those with social problems, for example, marital conflicts, were referred to the social worker or appropriate community resources. Close liaison was maintained with the attending obstetrician.

### 2.4. Outcome Measures

Measures were taken at (a) baseline, when women first engaged into clinical intervention with the psychiatrist and then (b) repeated at 6 months or at discharge, whichever was earlier. These included the EPDS (described earlier), the Global Assessment of Functioning Scale (GAF) [18], and the EuroQol EQ5D [19].

- The GAF is a 100-point scale that measures overall level of psychological, social, and occupational functioning on a hypothetical continuum and is particularly useful for managed care-driven diagnostic evaluations to determine the eligibility for treatment and disability benefits and to delineate the level of care required for patients.
- The EuroQol health index, EQ5D, is a generic measure of health-related quality of life that consists of a self-classifier and a visual analogue scale and can be used in the clinical and economic evaluation of health care and in population health surveys. The self-classifier consists of 5 items assessing health in 5 dimensions of mobility, self-care, usual activities, pain/discomfort, and anxiety/depression on a 3-point Likert scale. The visual analog scale is a vertical, graduated thermometer from 0 (worst imaginable health state) to 100 (best imaginable health state).
- We also administered a patient satisfaction survey at discharge: a 5-point Likert scale we developed to ask participants three questions, whether they were satisfied with medical care, counselling support, and psychoeducation provided about postpartum depression. Satisfaction was determined by a response ranging from “very dissatisfied” to “very satisfied”.

### 2.5. Data Analysis

Data in numbers, percentages, means, and standard deviations were presented as descriptives. For the scale/interval ratio variables, independent *t*-test (normality of data) or Mann-Whitney *U* test (nonnormality of data) was used to compare mean scores. For variables that involved categorical data, Pearson's chi-square was used. We used the Kruskal-Wallis test when comparing the EPDS scores category of the four race categories. Analyses were conducted with SPSS version 14.0.

## 3. Results

2148 postpartum women presented at the selected clinics, and 64% (1367) participated in screening and received educational information about postnatal depression.

### 3.1. Characteristics of Women Who Participated in the Screening Programme

Two-thirds (71%) of the women who participated in the screening programme were aged 25 to 34 years, with mean age 30.52 years (SD = 5.00), range from 18 to 44 years old. Half of the participating women were Chinese (52.1%), with Malays (19.2%) and Indians (14.2%) forming one-third of the sample, whilst 14.5% were of other races. 56.5% were Singapore-born, whilst 43.5% were foreign-born. The majority of women (94%) lived in public housing, whilst 6% lived in private housing.

### 3.2. Screening Results

Of the 1367 women screened, the majority (85%) scored below 10 on the EPDS, with 6% (80) having borderline scores of 10–12 (see Figure 1). Nine percent (126) were assessed to have EPDS score of 13 or more, indicative of probable postpartum depressive illness. The mean EPDS score of the cohort was 5.22 (SD = 4.87).

Pearson's chi-square tests compared the EPDS scores category between Singaporean and foreign-born migrant women (Table 2), and there was a statistically significant greater proportion of Singaporean women that scored above the cutoff of 13 or more than non-Singaporeans (*P* < .05).

Looking at the comparison across races (Table 3), the respective prevalence rates of high EPDS scores were seen in 9% (64) of Chinese, 7% (19) of Malays, 15% (28) of Indians, and 4% (9) of other races. Using the Kruskal-Wallis test, there was a statistically significantly greater proportion of Indian women who scored above the cutoff (EPDS 13 or more) than the other races, (*χ* ^2^ (3, *N* = 1367) = 15.502, *P* = 0.001).

There was no difference in terms of EPDS scores when comparing between those who lived in private housing with those who lived in public housing.

### 3.3. Characteristics of Women Entering The Intervention

Only 32.5% (41) of the 126 women with high EPDS scores accepted clinical intervention. The others declined referral due to various reasons, such as no time, cost concerns, stigma of receiving psychiatric disorder, no insight, and other reasons. 2 foreign-born women left Singapore.

The characteristics of the 41 women who entered intervention are summarized in Table 4.

Sixty percent (24) were Chinese, 95% (39) married, with 83% (34) having at least secondary-level or high-school-equivalent education, and 66% (23) were working. Interestingly, two-thirds (22) had not planned their pregnancy, and this was the first pregnancy for almost two-thirds of the women (25). One-third (14) had other children below the age of 5. Thirty-nine percent (16) had no available help whilst the others had help from live-in domestic help, relatives, or nursery.

### 3.4. Clinical Diagnoses of Intervention Group

Of the 41 women who engaged in clinical intervention, diagnosis was made based on DSM-IV criteria [20]. 44% (18) were found to suffer from major depression (postpartum onset), whilst 24.4% (10) had major depression (antepartum onset, currently postpartum), and 17% (7) had minor depression (postpartum onset). The rest received diagnoses including anxiety disorder (postpartum onset), adjustment disorder with depression (postpartum onset), acute grief reaction, dysthymia, minor depression (antepartum onset, now postpartum). 7 women had secondary diagnoses, such as underlying posttraumatic stress disorder (PTSD) from the birth experience, obsessive compulsive disorder (OCD), anxiety disorder.

### 3.5. Outcomes of Clinical Intervention

**Figure 2:**
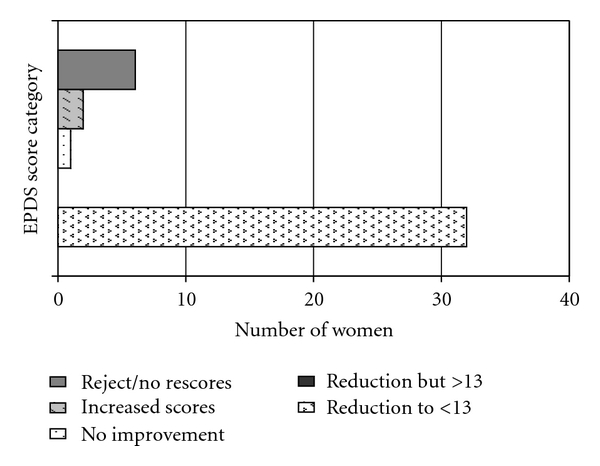
Change in EPDS scores with intervention.

**Figure 3:**
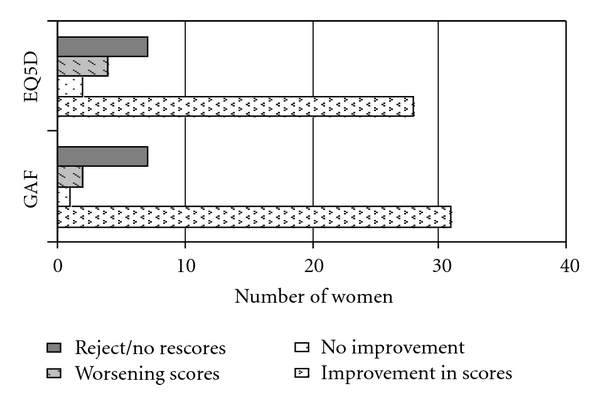
Change in GAF and EQ5D scores with intervention.

Of these 41 under clinical intervention, Seventy-eight percent (32) experiencing a reduction of scores to below the cut-off score of 13. 2 patients had increased scores, and 1 had no change (Figure 2). 76% (31) had reduction in GAF scores, indicative of improvement in function and symptoms, whilst 68% (28) had reduction in EQ-5D utility scores, indicative of improvement in the health status (Figure 3). Three patients had either no-change or increased scores on the EPDS, GAF, and EQ-5D measures—they had concurrent social issues that contributed to their ongoing distress, particularly ongoing marital conflicts and lack of social support.

### 3.6. Outcomes of Support and Counselling

**Figure 4:**
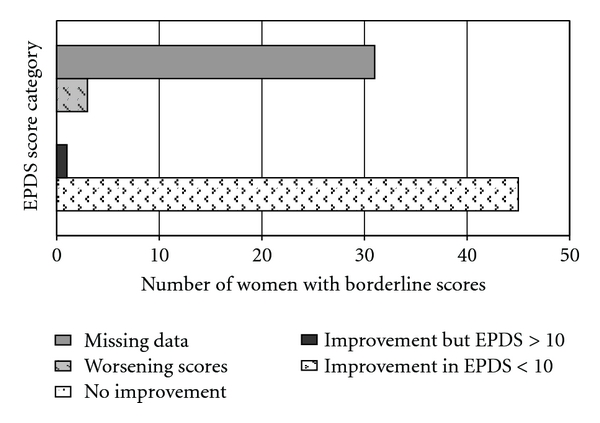
Change in EPDS scores amongst women who scored borderline (EPDS 10–12).

**Figure 5:**
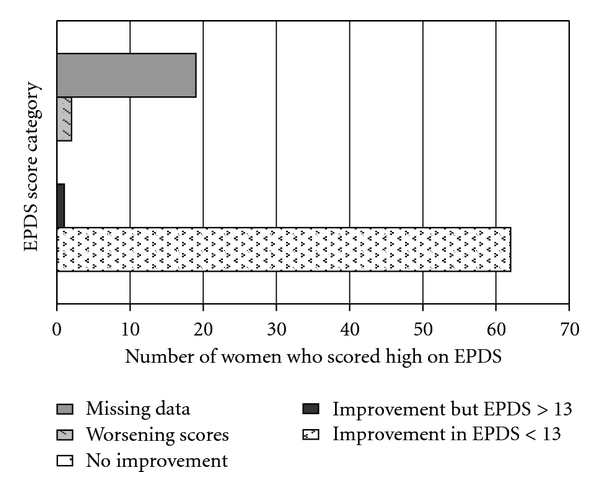
Change in EPDS scores amongst women who scored high (EPDS ≥ 13).

Together with the 67% (84) of high scorers who rejected intervention, 80 women with borderline EPDS scores (10–12) were provided supportive counselling by the case managers. Repeating EPDS showed that 74% (62) of high scorers and 56% (45) of borderline scorers benefited from this, with reduction in the EPDS scores (Figures 4 & 5).

### 3.7. Comparison between High Scorers Who Accepted Intervention and those Who Rejected Intervention

Those who accepted clinical intervention presented significantly later in the postpartum period, compared to those who declined (8 weeks versus 4.3 weeks) (*P* < .05).

Those who declined clinical care had lower mean baseline EPDS score compared to the high scorers who accepted intervention (15.8 versus 19.1), with this difference being statistically significant (*P* < .05). 

In terms of improvement in the EPDS symptom scores, the mean difference between those who entered intervention was 12.9, compared with 10.7 for those who declined clinical care, and this was statistically significant (*P* < .05).

### 3.8. Patient Satisfaction with Screening and Clinical Intervention

More than 95% of the women who participated in the screening programme were either satisfied or very satisfied with the counselling support and psychoeducation. Whilst 71% of those who entered clinical intervention were either satisfied or very satisfied with the medical care, and the rest felt neutral, whilst none were dissatisfied.

## 4. Discussion

9% of our cohort of 1369 postpartum scored above the cutoff on the EPDS, indicative of possible caseness, reflecting worldwide prevalence rates of postpartum depression [21, 22]. The finding that more Singaporean women compared to foreign-born women had higher scores on EPDS (10% versus 7%) is surprising, as we had expected similar findings as Buist et al. [8]—that migrant women face more challenges, and therefore, are more likely to be depression. Perhaps one explanation for the difference could be that Singaporean women tend to be from a less advantaged socioeconomic background as reflected by a smaller proportion living in private housing (4%), which is a proxy measure for socioeconomic status, as compared to foreign-born women (8%) delivering at KKH. However, there was no difference in the mean EPDS scores when comparing housing types. Further study is warranted, especially looking at cultural factors.

Notably, 66% of high scorers declined psychiatric intervention even with the well-trained case manager engaging and reassuring them about seeking help. The stigma of a psychiatric disorder can be significant, especially in the context of motherhood. Indeed, it is well established that one of the most challenging barriers related to help seeking for mental-health-related problems is stigma [23]. Ongoing efforts to destigmatize postpartum depression through public education and enhancing awareness is imperative [24] as is further research into help-seeking behaviour in postnatal depression.

Additionally, it is likely that women who declined intervention experienced less symptoms, as reflected by lower mean baselines EPDS scores and were thus less distressed. Results also demonstrated that those who accepted intervention presented later, possibly reflecting a longer duration of distress although the onset of symptoms was not studied. 

The main limitation of this research is that it is non-randomized and observational; hence, the groups are not directly comparable and results not generalizable.

The results demonstrate that the majority (three-quarters) of the depressed mothers who accepted treatment responded well to the intervention. Social risk factors, particularly marital conflicts and lack of social support, were seen in the women who experienced no improvement or worsening outcomes. Indeed, depressed patients with greater dyadic (marital) discord have been found to have a lower likelihood of remission of their depression during medication treatment [25]. 

Both the screening and intervention programme were well accepted, with more than 95% and 71% patient satisfaction, respectively. 

With no previous existing screening programme in Singapore, we believe that many women have struggled or presented late when the impact of the illness would have set in. With this programme, we now have the means to address the maternal mental health needs of our women. Indeed, with a foreign-born population representing almost half of our cohort, with nationalities or races from many countries, this model can be applicable to a wider global community.

## 5. Conclusions

Postpartum depression is a significant public health problem, but with a right-tier approach integrated on-site into obstetric setting, outcomes can be improved, and this model of care can be replicated.

##  Conflicts of Interest

The authors declare that there is no conflict of interests.

## Figures and Tables

**Table 1:** Supportive Therapy and Counselling In Perinatal Depression.

(I) Individual care
Early phase
(i) *Setting the therapeutic relationship *
Establishing rapport
Developing therapeutic alliance
(ii) *Empathic listening *
Encouraging expression of emotion and thought
Clarify thinking
Empathic mirroring and validation
Support, reassurance, encouragement
(iii) *Problem solving *
Exploring problems, possible solutions
(iv) *Psychoeducation *
Advise about illness and possible causative factors
Counselling about treatment options
Counselling about expected progress
Mid phase
(v) *Supportive therapy in dealing with individual issues *
(a) addressing the mother's self-percept,
for example, dealing with negative self-view
(borrowing from CBT),
(b) Addressing role changes (borrowing from
interpersonal therapy),
(c) Issues related to unwanted pregnancy, past trauma,
precious pregnancy, and so forth.
Recovery Phase
(vi) *Psychoeducation *
Advise about future risks
Counselling regarding long-term treatment (maintenance
options discussed, if needed)
(vii) *Empowerment, rebuilding of self *
Enhancing strengths, positive encouragement
Instilling hope, empowering woman as mother

(II) Care engaging husband/partner
(i) *Psychoeducation *
Advise about illness, treatment options
Advise about risks to self/fetus or infant
(ii) *Counselling to enhance support to patient *
Addressing areas of need
Facilitating the understanding of illness
Encouraging support
(iii) *Brief assessment of needs of husband/partner *
Brief exploration of husband's/partner's coping
Brief exploration of needs and counselling on resources
available

**Table 2:** Comparison of EPDS scores between Singaporean and non-Singaporean women.

Nationality	EDPS ≥ 13	EPDS < 13	*N*
Singaporeans	11%**	89%	773
Non-Singaporeans	7%	93%	594

**Pearson's Chi-square statistical significance at *P* < 0.05.

**Table 3:** Comparison of EPDS scores category between races.

	Chinese	Malays	Indians	Others	*N*
EPDS score < 13	91	93	85	96	1247
EPDS score ≥ 13	9	7	15**	4	120

**Kruskal-Wallis test statistical significance at *P* = 0.001.

**Table 4:** Descriptives of women entering intervention.

	Number	Percentage (%)
Maternal age at birth		
18–24 years	4	10
25–34 years	29	71
35–40 years	7	17
>41 years	1	2
Race		
Chinese	24	59
Malay	8	20
Indian	8	20
Others	1	2
Marital status		
Married	39	95
Single	1	2
Divorced	0	0
Separated	0	0
Cohabiting	1	2
Educational qualification at enrolment		
Primary	1	2
Vocational	6	15
Secondary	9	22
Tertiary	14	34
Degree	11	27
Occupation		
Professional executive/senior Management	8	20
	3	7
General administrative/supervisory	5	12
Service line	7	17
Self-employed/business owner	0	0
Home maker/unemployed	18	44
Pregnancy details		
Planned pregnancy	14	34
Unplanned pregnancy	27	66
Breast feeding	24	59
Had no other child	25	61
Had other children under 5 years old	41	100
Had termination(s) before	7	17
Had miscarriage(s) before	6	15

## Appendix

1. I have had thoughts of harming my baby.

   □ Yes      □ No

(2) I have heard voices when no one was around.

   □ Yes      □ No

(3) I have felt people around me wish to harm me, or have bad intentions.

   □ Yes      □ No
